# Blood-based biomarkers and neuroimaging for early detection of post-stroke cognitive impairment: current evidence and synergistic prospects

**DOI:** 10.3389/fneur.2025.1596940

**Published:** 2025-08-06

**Authors:** Ping Li, Yue Xin, Chunxiao Li, Laisong Yao, Yuekang Su

**Affiliations:** ^1^Department of Neurology, The Fifth Affiliated Hospital of Kunming Medical University, Yunnan Honghe Prefecture Central Hospital (Ge Jiu People's Hospital), Gejiu, China; ^2^Department of Outpatient, The Fifth Affiliated Hospital of Kunming Medical University, Yunnan Honghe Prefecture Central Hospital (Ge Jiu People's Hospital), Gejiu, China

**Keywords:** post-stroke cognitive impairment, blood-based testing, biomarkers, neuroimaging, diagnosis

## Abstract

Post stroke cognitive impairment (PSCI) is a series of common complications caused by stroke, ranging from mild cognitive impairment to dementia, which seriously affects the recovery and living quality of patients. Currently, the diagnosis of PSCI in the clinic mostly relies on subjective scale assessment, the untimeliness and imprecision of results greatly limit the efficient identification as well as the subsequent diagnosis and treatment of PSCI. With the increasing popularity and optimization of bioassay techniques and equipment, more and more studies have identified potential early warning markers of stroke patients with the development of their cognitive deficits through hematological testing or imaging. Therefore, the application of blood-based biomarkers and imaging techniques is important for the early identification of PSCI. This review focuses on the research progress of the above two testing modalities in PSCI to discuss their vital meanings for disease recognition. It also suggests that the combined application of the two is expected to improve the potential value of early and accurate diagnosis, with a view to providing new ideas for the clinical diagnosis and treatment of PSCI.

## 1 Introduction

Stroke is the most common type of cerebrovascular disease. It affects up to 20% of the population in high-income countries and up to 50% in some low-income countries (1). The onset of a stroke not only causes damage to the patient’s physical functioning, but is often accompanied by a range of complications from mild cognitive impairment to dementia, which are known as post-stroke cognitive impairment (PSCI) (2). According to previous studies, stroke patients have at least a 5–8 times increased risk of developing cognitive impairment compared to the rest of the population (3). PSCI refers to a cognitive impairment syndrome that persists for 6 months after a stroke and meets diagnostic criteria (4). However, its clinical symptoms are often insidious, and even with a standard three-month follow-up evaluation, it is difficult to identify them in a timely and accurate manner (5). Cognition includes multiple vital functions such as perception, speech, memory, and behavioral decision-making. Hence, the negative impact of PSCI on individuals and society should not be ignored. Besides its own direct damage, PSCI also increases the overall difficulty in the prognosis and rehabilitation of stroke patients, thus slowing down the pace of patients returning to normal life (6).

The diagnosis of PSCI in clinical practice relies on the patient’s superficial symptoms and neuropsychological assessment. These approaches are easily confounded by factors such as age, language proficiency, and educational attainment. Consequently, the evaluation often lacks timeliness, objectivity as well as accuracy. With the constant progress and wide application of clinical testing and imaging technologies, the research on the use of hematological testing and imaging interpretation in the field of non-organic diseases, such as cognitive and mood disorders, is becoming more and more in-depth (7–9). Accumulating evidence indicates that, in patients with PSCI, expression levels of specific protein factors in the blood are tightly correlated with the severity of cognitive dysfunction, which may be a potential biomarker for early prediction of disease onset (10, 11). Meanwhile, neuroimaging techniques, including structural MRI and functional MRI, also play an important role in evaluating brain changes in patients with cognitive impairment (12). However, the objective diagnostic indicators and modalities for PSCI are still unclear. Therefore, finding reliable biomarkers and diagnostic tools is crucial in order to achieve early identification and timely intervention of PSCI.

In this review, we synthesize current evidence on the application of blood-based biomarkers and imaging techniques for the early recognition of PSCI. Following an overview of the respective advantages and limitations of each testing modality, we critically discuss how their integration strengthens the diagnostic accuracy of PSCI. This review aims to facilitate early detection and optimize the effectiveness of clinical management strategies for PSCI.

## 2 Search method

We searched the PubMed electronic database for articles related to this review and manually identified and included eligible references. We use the following keywords to search for all possible combinations: “stroke,” “post-stroke cognitive impairment,” “cognitive impairment,” “neurodegenerative diseases,” “blood-based testing,” “imaging technology,” “neuroimaging,” “disease diagnosis,” “predictive indicators,” and “biomarker.”

## 3 Epidemiology and pathophysiology of PSCI

### 3.1 Epidemiology

PSCI is an important subtype of vascular cognitive impairment and one of the most common long-term complications of stroke. Existing evidence suggests that the prevalence of stroke varies significantly depending on diagnostic criteria, evaluation time points, and population characteristics: an international multicenter cohort study reported an overall prevalence of 38% in the first year after stroke (13). In a large-scale cross-sectional survey covering 30 provinces in China, it was found that the prevalence of PSCI in elderly patients can reach as high as 78.7% (14). Although there is heterogeneity in incidence rate reported in different studies, for PSCI patients, the superposition effect generated by the interaction between cerebrovascular injury and cognitive degradation not only further aggravates the pathological process, but also challenges the effect of clinical treatment and reduces the effectiveness of intervention measures. The epidemiological data of PSCI once again highlights its global public health burden and its profound impact on the health of the elderly population (14). Early identification and precise intervention of PSCI have become key issues that urgently need to be addressed in post-stroke management.

### 3.2 Pathophysiology

The onset and progression of PSCI constitute a multifactorial and mechanistically intricate pathological process. Although current research is constantly deepening in an attempt to reveal its specific mechanisms, the exact pathophysiology of PSCI has not yet been fully clarified. According to existing research, the potential pathological basis of PSCI includes white matter damage, blood–brain barrier (BBB) disruption, neuroinflammation, cerebral hypoperfusion, and other pathological effects (15–19). Among them, white matter hyperintensity caused by cerebral vascular injury often indicates a decline in patients’ cognitive function, especially in executive function, attention, language, and visuospatial abilities (20). The BBB serves as the brain’s primary defensive shield. Once compromised, it permits circulating toxins and inflammatory mediators to penetrate the brain, amplifying neuronal and axonal injury and further leading to cognitive impairment (21, 22). In addition, neuroinflammation, as another important influencing factor of PSCI, the activation of glial cells and the release of inflammatory factors may cause neural death or dysfunction, thereby leading to the occurrence of cognitive impairment in patients (23, 24). These studies also imply the important application potential of detecting biological indicators and brain tissue morphological and functional changes to identify PSCI-related pathological changes. Therefore, we further summarize and discuss the research progress of serum and imaging techniques related to PSCI.

## 4 Blood-based biomarkers in PSCI

Blood-based testing, as a pivotal non-invasive clinical tool, enables the real-time monitoring of disease dynamics through the analysis of small volumes of patients’ blood samples. This method is highly sensitive in reflecting critical physiological and pathological processes such as neuronal injury, inflammatory response, and metabolic status in patients. Consequently, it provides a timely biological basis for assessing disease progression. As research on biomarkers deepens, their value in the diagnosis and prognosis of PSCI has been increasingly recognized and validated.

### 4.1 Neurodegenerative and neural injury markers

#### 4.1.1 Neurofilament light chain (NfL)

NfL is a neuronal-specific structural protein that is primarily located in neuronal axons. When neurons are damaged, it is released into the cerebrospinal fluid and blood. Therefore, the level of NfL in the serum will change to some extent after neuronal injury. In recent years, numerous studies have shown that the expression level of NfL is closely related to neurodegenerative diseases (25–27). Wu et al. (28) found that the serum NfL level in PSCI patients was significantly higher than that in the healthy control group, and it was positively correlated with the severity of cognitive impairment, indicating that NfL could be a potentially sensitive indicator for reflecting early neuronal injury and the progression of cognitive impairment. A large-sample prospective cohort study was also shown that the concentration of plasma NfL within 48 h after stroke is an independent risk factor for the occurrence of PSCI at 90 days after onset (29). These studies all suggest the high predictive value and specificity of NfL for PSCI, which warrants further exploration.

#### 4.1.2 Tau protein

Tau protein, a microtubule-associated protein highly expressed in the CNS, has been shown to play a key role in the pathogenesis of neurodegenerative diseases through its phosphorylation and aggregation (30–32). In a longitudinal study in 2022, it was found that the assessment of blood phosphorylated-tau181 levels can effectively predict the risk of PSCI at 3 and 12 months after stroke (33). Notably, Tau protein detected in peripheral blood is predominantly of non-neuronal origin. This underscores the importance of precisely identifying brain-derived Tau protein for predicting CNS disorders, thereby enhancing the clinical relevance and diagnostic potential of peripheral blood biomarkers. In this regard, Gonzalez-Ortiz et al. developed a highly sensitive detection method that selectively binds to brain-derived Tau protein, thereby avoiding the interference of the “big Tau” subtype expressed peripherally, and this blood-based brain-derived Tau protein detection method has been verified in multiple independent cohorts (34). Moreover, the researcher also demonstrated that serum BD-tau concentrations were on average 40% lower than those measured in matched plasma samples, underscoring the critical importance of standardizing specimen type when quantifying this biomarker (35). Although Tau protein is now established as a pivotal biomarker in Alzheimer’s disease (AD), evidence supporting its clinical utility in PSCI remains limited. Nevertheless, the pathophysiological overlap between PSCI and AD provides a compelling rationale for further investigation into the diagnostic and prognostic relevance of Tau in PSCI.

#### 4.1.3 *β*-Amyloid (aβ)

Aβ is a neuronal metabolite released at synapses. Its aberrant elevation serves as a key clinical biomarker for the identification and diagnosis of neurodegenerative diseases (36). An increasing number of studies have focused on the potential role of Aβ in PSCI (37, 38). A decline in plasma Aβ1-42 levels 3 months after stroke constitutes the most significant independent predictor of PSCI at 1 year (38). In a prospective survey by Kang et al., it was found that Aβ, produced by the enzymatic cleavage of amyloid precursor protein, is an important predictor of the development of PSCI and cognitive decline over 1 year (39). Although the determination of Aβ levels in the blood has the advantages of being minimally invasive, rapid, and economical as a biomarker for PSCI, its clinical application is still challenged by BBB, the low concentration of Aβ in the blood, and potential interference from other proteins in the blood (40). Against this background, positron emission tomography (PET), as an emerging imaging technique, provides a new perspective and method for the clinical assessment of Aβ deposition and Tau protein (39, 41).

#### 4.1.4 Brain-derived neurotrophic factor (BDNF)

BDNF is a key neurotrophic protein in the CNS, playing an important role in brain activities such as learning, memory, comprehension, and injury repair (42). BDNF exerts its biological functions by binding to its specific receptor tropomyosin receptor kinase B (TrkB) and subsequently activating downstream signaling pathways. This cascade of events enables BDNF to perform a variety of critical biological functions, including enhancing synaptic plasticity, promoting neurogenesis, and supporting cell survival (43). Previous research has demonstrated that serum BDNF levels are significantly diminished in patients with dementia, with severe cognitive impairment being closely associated with reduced BDNF concentrations (44). Additionally, stroke patients have been found to exhibit a significant decrease in serum BDNF expression (45). These findings collectively suggest that BDNF may serve as a potential biomarker for evaluating the risk of PSCI. In a clinical study by Chang et al., it was shown that elevated serum BDNF levels are associated with a lower risk of PSCI at 3 months (46). In previous animal studies on PSCI treatment, the reduction of BDNF expression in brain regions such as the hippocampus of rats can be reversed (47, 48). In summary, the relationship between BDNF levels and PSCI is not only in its potential as a biomarker but also in its positive effects on neuroplasticity and cognitive function. This makes BDNF a promising therapeutic target for PSCI.

#### 4.1.5 Others

In addition to the aforementioned neuro-related biomarkers, recent studies have also proposed several potential biomarkers for predicting PSCI and other neurogenic cognitive injuries. Neuron-specific enolase (NSE) is an enzyme highly expressed in neural tissue and is a biochemical biomarker for neuronal injury (49). In a study by Hu et al., NSE was also used as one of the parallel reference indicators for the prognosis assessment of PSCI treatment (50). Furthermore, glial cells, as key immune effector cells in the CNS, play a multifaceted role in the occurrence and development of PSCI. They participate in the pathological process of PSCI by regulating neuroinflammatory responses and affecting the repair and regeneration of neural functions (51).

These biomarkers are not only critical for the diagnosis and prognosis assessment of PSCI but also provide key references for revealing the molecular mechanisms of PSCI. However, when detecting neuro-related biomarkers through serum methods, there is a challenge of source specificity, which requires precise distinction and quantification of biomarkers derived from the CNS.

### 4.2 Immune and inflammatory markers

#### 4.2.1 C-reactive protein (CRP)

CRP is a hepatically derived pattern recognition molecule that exhibits a marked increase in response to inflammatory stimuli. Owing to its sensitivity and rapid elevation during inflammatory states, CRP serves as a widely utilized biomarker for systemic inflammation in clinical practice (52). Previous studies have proposed an association between brain inflammation and cognitive decline in neurodegenerative diseases (53, 54). Meanwhile, the expression levels of CRP in patients with cognitive impairment and dementia are closely related to cognitive decline (55). Baba et al. were found that within the first 3 months after stroke, the prevalence of cognitive impairment and serum CRP levels in patients were both higher, and high CRP levels were associated with the duration of stroke and working memory domain of cognition (56). In another follow-up study lasting 10 years, it was shown that there is a significant correlation between CRP concentration and long-term cognitive decline (57). Moreover, in the analysis of risk factors for cognitive impairment in stroke patients, CRP may be an important independent predictor of PSCI (58). Although the detection of CRP is widely used in clinical practice due to its convenience and sensitivity, its specificity for non-inflammatory dominant diseases is limited. Therefore, possible strategies to address this issue include: first, combining CRP with other biomarkers that have higher specificity or alternative detection methods to enhance the accuracy of disease prediction; and second, clarifying the expression threshold or range of CRP in specific disease contexts to better interpret its significance.

#### 4.2.2 Interleukin (IL)

IL is a class of multifunctional cytokines that can be produced by various cells and play a key role in immune and inflammatory responses, regulating the activity and function of immune cells in the body. Numerous previous researches have found that the expression of IL-6, IL-8, IL-10, and other members of the IL family is closely related to the cognitive function of stroke patients (59–62). In a study by Feng et al. (63) the plasma IL-6 level in PSCI patients was higher than that in the cognitively normal group and was significantly negatively correlated with the Montreal Cognitive Assessment score. This was also confirmed in a 2022 study (64). In addition, IL-10 were elevated in the serum of patients with cognitive impairment related to executive function, while it shows a decreasing trend in cerebrospinal fluid (65). A five-year follow-up research was shown that elevated serum IL-8 levels after ischemic stroke were independently associated with baseline cognitive impairment, while elevated serum IL-12 levels were associated with subsequent cognitive impairment (66). In summary, ILs not only participate in inflammation and immune responses but may also be directly related to cognitive decline. These findings provide a crucial foundation for considering ILs as potential blood biomarkers for PSCI. Moreover, they offer a new direction for the early diagnosis and treatment of PSCI, as well as for exploring its underlying pathological mechanisms.

#### 4.2.3 Rheumatoid factor (RF)

RF is an autoantibody commonly found in rheumatoid arthritis, and its potential role in PSCI has gradually attracted attention in recent years. The positive expression of RF is associated with an increased risk of poor outcomes after ischemic stroke (67). Zhu et al. (68) was also found that elevated serum RF levels in the acute phase of ischemic stroke were independently associated with cognitive impairment at 3 months. Additionally, the combination of RF and other biomarkers has also been confirmed to improve the risk prediction of PSCI (69). However, there are still challenges in using RF as a biomarker for PSCI. On the one hand, the specificity and sensitivity of RF in PSCI have not yet been fully verified, and its diagnosis in PSCI may be influenced by multiple factors such as age or gender. On the other hand, the precise mechanism of action of RF in PSCI remains unclear. Further basic and clinical research is needed to elucidate these aspects.

#### 4.2.4 Triggering receptor expressed on myeloid cells (TREM)

TREM is a family of novel pattern recognition receptors widely expressed in various immune cells. The TREM family includes TREM-1, TREM-2, TREM-3, etc., which mediate cell signal transduction by recognizing different ligands and thereby participate in the functional regulation of cells. Among them, the role of TREM-2 in stroke and PSCI has attracted much attention. In a large-sample community survey in Japan, the elevated serum TREM-2 levels are significantly associated with neurodegenerative diseases in the elderly population (70). It was further clarified that the increase in plasma TREM-2 has important potential value in predicting PSCI (71). TREM-2 is highly expressed in microglia in the CNS and can also mediate inflammatory responses in microglia to intervene in cognitive function in mice (72). In addition, TREM-1, which is also highly expressed in microglia, can participate in neuroinflammation (73). The above studies also suggest a close link between PSCI and neuroinflammation, and it is worth exploring whether TREM-1 is also associated with PSCI.

Naturally, in addition to detecting the above single immune and inflammatory factors in the blood, the routine blood-based test commonly used in clinical practice can also efficiently reflect the body’s immune and inflammatory conditions, and the related detection indicators involved in it also have a certain degree of predictive effect on PSCI, such as the neutrophil-lymphocyte ratio (NLR) and the HALP score (hemoglobin, albumin, lymphocytes, and platelets) (74–76).

### 4.3 Oxidative and metabolic markers

#### 4.3.1 Homocysteine (Hcy)

Hcy is an important intermediate product in the methionine metabolic process and is commonly used in clinical practice for risk assessment of cardiovascular and cerebrovascular diseases (77). In previous studies, a close relationship between Hcy and PSCI has been found (78, 79). In a study by Zhou et al., it was found that elevated Hcy levels in the acute phase of ischemic stroke are independently associated with subsequent cognitive impairment, and this association is more significant in younger patients (under 65 years old) (80). In addition to age differences, there may also be gender differences in the relationship between Hcy and PSCI. A prospective cohort study was found that elevated homocysteine levels increase the risk of PSCI at 12 months in women, but not in men (81). Furthermore, high-frequency repetitive transcranial magnetic stimulation (HF-rTMS) combined with galantamine treatment for PSCI can effectively reduce serum Hcy and improve patients’ cognitive function (50). Moreover, the deficiency of B vitamins is also related to cognitive decline and may be associated with hyperhomocysteinemia caused by insufficient vitamin B (82). However, whether vitamin B supplementation is beneficial for the improvement of PSCI is still controversial (83–85).

#### 4.3.2 Trimethylamine N-oxide (TMAO)

As an emerging potential predictor in cardiovascular and neurological diseases, TMAO, produced by gut microbial metabolism, has also been found to play an important role in the occurrence and development of cognitive impairment (86). Elevated serum levels of TMAO are associated with an increased risk of ischemic stroke and more severe neurological deficits, highlighting its potential as a biomarker for screening moderate-to-severe stroke (87). Furthermore, in a risk factor analysis, these levels have been identified as an independent predictor of PSCI (88). With the rapid progress in the field of “gut-brain axis,” studies have begun to explore the potential interactions and pathogenic mechanisms between gut metabolites such as TMAO and cognitive function (89). Tu et al. summarized that TMAO may promote the progression of stroke and the occurrence of cognitive impairment by regulating cholesterol metabolism, foam cell formation, and platelet hyperreactivity (86). Hence, monitoring TMAO levels may become a potential biomarker for the early identification and intervention of PSCI.

#### 4.3.3 Gamma-glutamyl transferase (GGT)

GGT is a serum metabolic biomarker commonly used in clinical practice to assess liver function and reflects the state of oxidative reactions in the body. In a multicenter prospective cohort study by Li et al., the serum GGT levels were negatively correlated with the risk of PSCI, and extremely low levels of GGT may also be a suspected risk factor for PSCI (90). Intriguingly, bidirectional two-sample Mendelian randomization analyses further indicate a causal relationship between genetically predicted GGT levels and stroke (91). Another study has also shown that higher expression levels of GGT are associated with cognitive decline in later life and the occurrence of vascular dementia (92). Moreover, the correlation between GGT and cognitive function exists in both men and women (93). The expression level of GGT shows dynamic changes at different stages of the disease, which may lead to variability in test results at different time points. To use GGT as a predictive biomarker for PSCI, future research should further explore the dynamic changes in GGT expression and precisely define the thresholds for its use in diagnosis and prognosis.

#### 4.3.4 Uric acid (UA)

UA, as the final product of purine metabolism, is usually considered an important indicator for assessing kidney function. With the continuous exploration of UA, its role in aging and neurodegenerative diseases has also attracted the attention of researchers (94–96). The increased serum UA levels were significantly positively correlated with lower cognitive scale scores in stroke patients and an increased risk of moderate to severe cognitive impairment 1 month after stroke (97). A meta-analysis conducted by Yan et al. demonstrated that elevated serum UA levels in patients with acute ischemic stroke may serve as a potential marker for increased risk of PSCI (98). In a clinical study by Xu et al., it was shown that elevated serum UA levels may be an independent risk factor for PSCI (99). Specifically, serum UA levels exceeding 363.58 μmol/L were found to have significant clinical relevance for predicting PSCI. From a pathological perspective, the increase in UA may be related to endothelial dysfunction and the occurrence of oxidative stress reactions (100, 101). Whether controlling elevated UA levels can mitigate the risk of PSCI remains to be determined through additional research and clinical trials.

In summary, blood-based testing technology plays a vital role in clinical disease assessment due to its convenience, high sensitivity, and specificity. Utilizing PSCI-specific biomarkers or combining specific thresholds of routine clinical indicators holds significant potential for predicting the early occurrence of disease, thereby offering substantial clinical value. Spurred by rapid advances in multi-omics technologies, profiling divergent molecules—including transcriptomic, proteomic, and metabolomic entities—in peripheral blood has emerged as a potentially key strategy for discovering PSCI biomarkers (102). Although numerous candidate biomarkers have been proposed (see in Table 1), their translation into widespread clinical practice remains contingent on resolving several key challenges, including the specific identification of molecular sources, the reproducibility of indicators, and the need for large-scale, multicenter studies to validate their efficacy. Future research should focus on addressing these key issues to advance the clinical application of serum biomarkers in PSCI prognosis.

**Table 1:** The key blood-based biomarkers of PSCI.

Biomarker	Sample source	Relationship with PSCI	
		Expression change	Measurement time
Aβ	Serum/Plasma	Decrease	3 months after stroke
BDNF	Serum	Decrease	3 months after stroke
CRP	Serum	Increase	The first 3 months after stroke
GGT	Serum	Extremely low levels	Unknown
Hcy	Serum	Increase	24 h after stroke
IL-6	Plasma	Increase	Unknown
IL-8/10/12	Serum	Increase	3 months after stroke
NfL	Serum/Plasma	Increase	48 h after stroke or within 3 months of stroke
RF	Serum	Increase	Acute Phase of Ischemic Stroke
Tau	Plasma	Decrease	Within 7 days of stroke or 3 months after stroke
TMAO	Serum/Plasma	Increase	24 h after stroke
TREM	Serum	Increase	Unknown
UA	Serum	Increase	Unknown

Aβ, Amyloid-beta peptide; BDNF, Brain-derived neurotrophic factor; CRP, C-reactive protein; GGT, Gamma-glutamyl transferase; Hcy, Homocysteine; IL, Interleukin; NfL, Neurofilament light chain; RF, Rheumatoid factor; TMAO, Trimethylamine N-oxide; TREM, Triggering receptor expressed on myeloid cells; UA, Uric acid.

## 5 Imaging techniques in PSCI

PSCI is a complex neurodegenerative disease, often characterized by hypoperfusion in specific brain regions and alterations in functional and structural architecture. These changes provide key biological markers for the imaging diagnosis of PSCI. Imaging techniques, with their non-invasive nature, real-time monitoring capabilities, and operational convenience, have become a routine auxiliary diagnostic tool in clinical practice. With the continuous progress of imaging technologies, their importance in the early prediction, therapeutic prognosis assessment, and risk stratification of PSCI is increasingly highlighted.

### 5.1 Structural imaging

Computed tomography (CT) and magnetic resonance imaging (MRI) are important structural imaging techniques that can help clinicians promptly and efficiently observe subtle changes in brain anatomy. As key tools for the diagnosis and dynamic monitoring of stroke, structural imaging techniques can accurately locate the lesion areas and the size of infarcts in patients (103, 104). With the in-depth exploration of brain structural changes associated with PSCI, these changes have gradually been identified through CT and MRI, which are considered potential biomarkers for predicting the occurrence of PSCI.

CT scanning, with its speed, convenience, and widespread availability, plays an vital role in the evaluation of acute stroke patients. In a meta-analysis by Ball et al., the identification of white matter lesions, cerebral cortical atrophy, and pre-existing stroke lesions by CT is closely related to the risk of PSCI (105). Among them, the presence of white matter lesions was found to increase the risk of PSCI by three times, and when combined with cortical atrophy, it is associated with the occurrence of dementia. In subsequent studies, it was also found that identifying cortical atrophy through CT can effectively assess the prognosis of PSCI (106). In addition, the efficient identification of intracranial vascular calcification and other small vessel lesions by CT can be beneficial for PSCI risk prediction (107). Although CT is extremely sensitive to acute cerebral hemorrhage, it may not always detect brain abnormalities in patients with hyperacute cerebral infarction.

Compared with CT, MRI, with its high resolution and sensitivity to soft tissues, can provide physicians with more detailed brain structures in neurological diseases. Zhong et al. (108) used MRI technology to detect not only white matter lesions, brain atrophy, and cerebral microbleeds in patients but also the damage to the intracranial cortical cholinergic pathway and found that it is related to cognitive impairment in stroke patients at 3 months. A 2025 study introduced a prognostic model that integrates MRI radiomics, quantitative electroencephalography, and clinical variables to predict cognitive impairment 1 year after acute ischemic stroke, thereby facilitating early risk stratification and precision management (109). Previous study was also found through MRI that in patients with basal ganglia infarction, remote brain atrophy and disconnection between the frontal lobe and the infarcted area have an essential impact on cognitive impairment (110). Moreover, Diffusion tensor imaging (DTI), an emerging imaging method developed based on MRI technology, has also been used to predict cognitive performance after stroke (111). Evaluating lymphatic system function based on this technology may also serve as a potential predictive indicator for PSCI (112).

### 5.2 Functional imaging

The development of functional imaging techniques has not only enabled the detection of physical structural changes in the brain but also provided clinicians with a crucial tool for assessing its pathological and physiological state (113). Functional MRI, which reflects brain function through blood oxygen level dependent signal fluctuations, serves as a sensitive biomarker for PSCI by quantifying regional activation and functional connectivity alterations within cognition-relevant networks (8, 114). This was also verified in a study by Miao et al. (115). Based on resting state functional MRI, Han K et al.’s study revealed the potential neural mechanisms of PSCI from a mechanistic perspective (116). In addition, Hoffmann M proposed that Single-photon emission CT (SPECT) is more sensitive than structural neuroimaging techniques in identifying PSCI (117). Previous research has also found through SPECT that there are perfusion abnormalities in the brains of patients with cognitive impairment after thalamic stroke (118). Besides, positron emission tomography (PET) can assess brain glucose metabolism using radioactively labeled glucose analogs (such as 18F-FDG). The technique facilitates the identification of metabolic disorders linked to cognitive impairment and provides insights into the relationship between neuroinflammation and amyloid deposition in PSCI (119). Deep learning models based on PET data have also been used to identify objective biomarkers of cognitive impairment in patients with cerebrovascular disease (120).

In conclusion, both structural and functional imaging techniques have been increasingly recognized for their value in assessing PSCI. Their combination can provide a more comprehensive brain network perspective for patient diagnosis and treatment. With the progress of imaging techniques and the development of analytical methods, their application prospects in the diagnosis and research of neurological diseases will become even broader.

## 6 Synergy between blood-based biomarkers and neuroimaging techniques

PSCI is a complex pathological process involving multidimensional clinical pathological changes and the interaction of numerous factors. Both blood-based testing from the molecular information level and neuroimaging assessment from the perspective of brain function and structural changes have demonstrated their unique advantages and significance. In previous study, it was pointed out that changes in different blood indicators are correlated with different characteristics of neurodegenerative diseases (121). Kulesh et al. (60) also demonstrated that cognitive performance in PSCI tightly correlates with both cytokine signatures and structural–functional brain alterations, and that their synergistic integration may form a more powerful and informative biomarker panel for predicting PSCI. The combination of the two types of detection indicators seems to be a more efficient informational biomarker for predicting PSCI. Similarly, compared with single-indicator assessment, the combination of serum NfL levels and brain infarct volume and white matter hyperintensity detected by imaging has better predictive value for PSCI (122). In recent years, researchers have successfully identified potential biomarkers and therapeutic targets for ischemic stroke by integrating neuroimaging, behavioral studies, and proteomic information from non-human primates (123).

In most cases, the expression levels of blood-based biomarkers can reflect the immediate state of the disease, and their changing trends can predict the progression of the disease (124). At the same time, neuroimaging techniques show significant advantages in tracking long-term changes in brain structure and function (125). The dynamic changes of these clinical indicators are crucial for predicting and assessing patients’ conditions, especially for asymptomatic patient groups. Therefore, finding timely and accurate biomarkers is key to early disease prediction. Although combining blood-based biomarkers and neuroimaging techniques could more comprehensively reveal the pathological mechanisms of PSCI, there are still many challenges in practice, including the selection of application indicators, standardization of techniques, and data analysis methods. To tackle these challenges, it is imperative to establish large-scale, multicenter databases. These repositories should efficiently aggregate and harmonize blood-based biomarker data with neuroimaging findings. Such an approach will significantly bolster the statistical robustness and broaden the applicability of the research findings.

## 7 Conclusion and future perspectives

**Figure 1:**
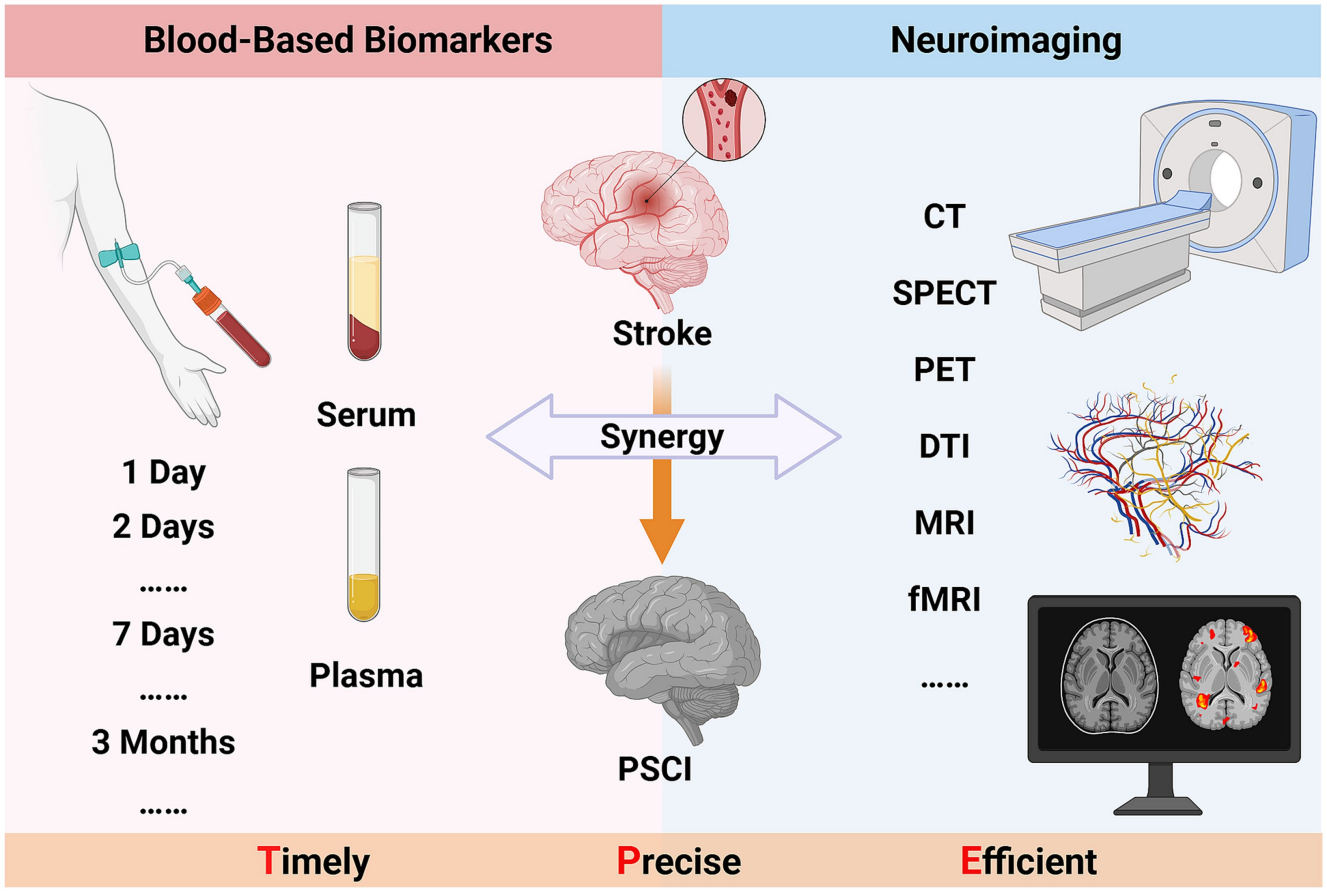
The synergy between blood-based biomarkers and neuroimaging techniques in PSCI (Created in https://BioRender.com).

The early and accurate identification of PSCI is of great significance for improving patients’ quality of life and clinical prognosis. However, current diagnostic methods generally suffer from insufficient objectivity and low efficiency. Hence, the application value of blood-based biomarker testing and neuroimaging techniques in the diagnosis of PSCI is increasingly prominent. Based on existing research findings, this review systematically summarizes the current application of these two auxiliary examination methods, fully explains their unique advantages, and the potential efficacy of their combined use. Starting from the biological molecular dimension, blood-based biomarkers can accurately assess the degree of neuronal injury, immune-inflammatory response, and oxidative metabolic levels related to the disease. Neuroimaging techniques, on the other hand, focus on the brain structure and function to meticulously monitor its microscopic pathological changes. Both provide key reference indicators for the early diagnosis and prediction of PSCI, greatly enriching clinical diagnostic information. The synergy between blood-based biomarkers and neuroimaging techniques represents an innovative opportunity in the clinical diagnosis and treatment of PSCI (see in Figure 1). However, it also poses new challenges to traditional diagnostic and treatment models. Future research must be systematically intensified to enhance the synergistic integration of technical platforms, expedite the iterative refinement of clinical diagnostic criteria, and mitigate the global disease burden of PSCI, thereby optimizing patient outcomes and quality of life.
